# 

**Published:** 1892-06

**Authors:** 


### PAMPHLETS RECEIVED.

Studies upon Injuries of the Kidneys, with Nephrolithotomy and Nephroraphy. By Aug. Schachner, M. D., Louisville, Ky.

Thirty-two Unselected Abdominal Sections. By Thomas Opie, M. D., Baltimore.

Pathology and Treatment of Tetanus. By D Braden Kyle, M. D., Philadelphia.

An account of the influenza as it appeared in Philadelphia in the winters of 1889–90, and of 1891–92. By J. Howe Adams, M. D., Philadelphia.

Tuberculin; the Value and Limitation of its Use in Consumption. By Charles Denison, M. D., Denver, Colorado.

Transactions of the Detroit Medical and Library Association, 1891. Dr. J. V. Becelaere, Librarian.

Trendelenburg's Posture in Gynecology. By Florian King, M. D., N. Y.

Total Extirpation versus Leaving a Stump, in Operation for Uterine Myomata. By Florian King, M. D., N. Y.

Bulletin of the Johns Hopkins Hospital, Baltimore, January, February, 1892.

Therapeutic Aspect of Some Ovarian Tumors. By Edw. W. Jenks, M. D., Detroit, Michigan.

Treatment of Laryngeal Phthisis. By Robert Levy, M. D., Denver, Col.

Indications for Colotomy. By Chas. B. Kelsey, M. D., New York.

Obstetric Problems; an Inquiry into the Nature of the Forces Determining Head Presentation, Internal Rotation, etc. By D. T. Smith, M. D., Louisville, Ky.

Neuroma, with report of a case. By E. J. A. Rogers, M. D., Denver, Col.

Contribution to Spinal Cord Surgery. By Archibald Church, M. D., Chicago, and D. W. Eisendrath, Chicago.

Tobacco, Insanity and Nervousness. By Dr. L. Bremer, St. Louis.

Aphasia due to Subdural Hemorrhage without External Signs of Injury. By Drs. L. Bremer, and N. B. Carson, St. Louis.

Gastrostomy. By N. Senn, M. D., Chicago.

Two Cervical Muscle Anorraties in the Negro. By Middleton Michel, M. D., Charleston.

